# Transient receptor potential ankyrin 1 (trpa1) mediates il-1β-induced apoptosis in rat chondrocytes via calcium overload and mitochondrial dysfunction

**DOI:** 10.1186/s12950-018-0204-9

**Published:** 2018-12-17

**Authors:** Songjiang Yin, Li Zhang, Liang Ding, Zhengquan Huang, Bo Xu, XiaoChen Li, Peimin Wang, Jun Mao

**Affiliations:** Departments of orthopedics, Affiliated Hospital of Nanjing University of TCM, Road Hanzhong 155#, Nanjing, Jiangsu Province, China

**Keywords:** Chondrocyte apoptosis, Osteoarthritis, Transient receptor potential ankyrin 1, Calcium overload, Mitochondrial dysfunction

## Abstract

**Background:**

Chondrocyte apoptosis is a central feature in the progression of osteoarthritis (OA), and would be triggered by sustained elevation of intracellular calcium ion (Ca^2+^), also known as a cellular second messenger. Transient receptor potential ankyrin 1 (TRPA1) is a membrane-associated cation channel, and the activation of which causes an influx of cation ions, in particularly Ca^2+^, into the activated cells. Therefore, we investigate the potential role of TRPA1 in mediating Ca^2+^ influx to promote chondrocyte apoptosis in OA.

**Methods:**

The expression of TRPA1 in interleukin (IL)-1β-treated rat chondrocytes was assessed by Polymerase chain reaction (PCR) and Western blot (WB), and the functionality of TRPA1 channel by Ca^2+^ influx measurements. Meanwhile, the chondrocyte apoptosis in IL-1β-treated cells was measured by TUNEL assay and flow cytometry. The measurement of mitochondrial membrane potential and apoptosis-associated proteins after inhibition of TRPA1 were also performed in IL-1β-treated rat chondrocytes.

**Results:**

After being induced by IL-1β, the gene and protein expression of TRPA1 was increased in the dose-dependent manner. Meanwhile, Ca^2+^ influx mediated by TRPA1 in rat chondrocytes was also enhanced. Pharmacological inhibition of TRPA1 downregulated the apoptotic rate in IL-1β-treated rat chondrocytes. In addition, the membrane potential depolarization was improved and significantly increased expression of apoptosis-associated proteins also reduced by the TRPA1 antagonist.

**Conclusions:**

We found the IL-1β caused the increased functional expression of TRPA1, the activation of which involved IL-1β-induced apoptosis in rat chondrocytes. The potential mechanism may be linked to the intracellular calcium overload mediated by TRPA1 and attendant mitochondrial dysfunction.

## Introduction

Knee Osteoarthritis (OA), the most common form of arthritis, is a major cause of joint pain, activity limitation, and physical disability in the elderly [1]. Pathologically, the disease is characterized by the progressive degeneration of articular cartilage [2]. Chondrocytes, the resident cells in articular cartilage, play an important role in the homeostasis of cartilage metabolism [3], and the compromising of chondrocyte function and survival would lead to the failure of articular cartilage [4]. In OA joint, the cartilage becomes hypocellular and lacunar emptying, which suggest the chondrocyte apoptosis is a central feature in the disease progression [5]. Therefore, the modification of chondrocyte apoptosis has gradually been considered as one of the potential therapeutic targets for OA [6].

Calcium ion (Ca^2+^) is a major intracellular second messenger considered as a key regulator of cell survival [7]. It is well known that sustained elevation of intracellular Ca^2+^ was capable of inducing Ca^2+^ entry-dependent ROS production [8], mitochondrial depolarization [9] and then apoptosis [10]. Transient receptor potential (TRP) ion channels are a large family of membrane-associated cation channels, which most permeable to Ca^2+^ [11]. Therefore, the activation of TRP channels serves as an important Ca^2+^ entry pathway contributing to fluctuation in intracellular Ca^2+^ and subsequent signal pathways [12]. Recently, emerging evidences also suggested the vital role of overload Ca^2+^ influx through TRP ion channels in cell deaths [13], such as hypoxia-induced apoptosis in H9C2 Cells [14], acidic solution-triggered apoptosis in synovial fibroblasts [15], and even monoiodoacetic acid (MIA) -mediated apoptosis in chondrocytes [16].

Transient receptor potential ankyrin 1 (TRPA1), a member of TRP ion channel, mainly mediates pain and inflammation [17], widely expressed in neuronal and non-neuronal cells [18]. The activation of TRPA1 causes an influx of Ca^2+^ into the activated cells, which has been shown to involve in biological processes as diverse as factors secretion [19], gene transcription [20] and cell death [21]. Recently, the effect of TRPA1 mediating synovial inflammation and cartilage degradation has been well recognized [22, 23], which was attributed mainly to a TRPA1-induced imbalance between the production of catabolic, anabolic, and inflammatory mediators [19, 24]. However, to date, no attention had been paid to define the relationship between TRPA1 and chondrocyte apoptosis. Therefore, we aimed to investigate whether or not the potential regulatory effect of TRPA1 in mediating IL-1β- induced apoptosis in rat chondrocytes.

## Methods and materials

### Isolation of rat chondrocytes

Rat chondrocytes were isolated as described [25]. In brief, the pieces of articular cartilage were aseptically dissected and separated from the tibial plateau. Then the cartilage was washed with phosphate-buffered saline (PBS) and cut into small pieces, which were digested in 0.2% collagenase type II for 6 h. After terminating digestion and filtrating, this suspension was then centrifuged at 1500 rpm for 6 min to collect the chondrocytes which cultured in a 10 cm^2^ culture flask (Corning, NY, USA) in DMEM supplemented with 10% fetal bovine serum (Gibco, MA, USA), 1% penicillin/ streptomycin (Invitrogen, CA, USA) at 37 °C in 5% CO^2^. All the experiments were conducted using rat chondrocytes from passages 2–3.

### In vitro treatment of rat chondrocytes

Rat chondrocytes were seeded in 2 mL of medium in a 6-well plate. Each culture was treated with IL-1β (PEPROTECH, USA) in the dose of 0 ng/ml, 0.1 ng/ml, 1 ng/ml, 5 ng/ml, 10 ng/ml for 12 h and 24 h respectively and incubated at 37 °C in 5% CO^2^. In addition, chondrocytes were also treated with IL-1β following pre-treated with the specific TRPA1 antagonist HC-030031 (Sigma, USA) at the dose of 100 μM which has been established previously [24], in order to detect apoptosis-associated proteins, mitochondrial membrane potential and apoptosis.

### Real-time PCR

RNA was isolated from treated chondrocytes with Trizol. RNA concentration and purity were measured by spectrophotometer, A260nm/A280nm ratio being 1.8~2.0. cDNA was synthesized from 2 μg of total RNA using random primers and M-MLV Reverse Transcriptase (Invitrogen, Carlsbad, CA, USA). RNA expression was measured with a SYBR Green PCR kit using ABI 7500 Real-Time PCR system (Applied Biosystems, CA, USA) according to the manufacturer’s instructions. The relative expression level of the target gene was calculated using the 2^-ΔΔCT^ method.

### Protein extraction and western blotting

Western blotting was performed as previously described [20]. Blots were incubated with primary antibodies including TRPA1 (Novus Biologicals, USA, dilution 2μg/ml), Bax (Proteintech, USA, dilution 1:1000), cytochrome c (Proteintech, USA, dilution 1:500), PARP1 (Proteintech, USA, dilution 1:500).

### Ca^2+^-influx measurements

Rat chondrocytes were loaded with 5 μM fluo-3-acetoxymethyl ester (Fluo-3-AM, Sigma, USA) and Pluronic F-127 (KeyGEN BioTECH, China) containing 25 mM HEPES (Sigma, USA) for 30 min at 37 °C in the dark, then washed twice with PBS to remove extracellular Fluo-3-AM. Imaging was performed using Leica inverted microscope and analyzed with ImageJ software. The measured average fluorescence intensity of each cell in the field (F) was normalized with the non-specific background fluorescence (F0) to obtain the fluorescence intensity (F/F0).

### Caspase-3 and Caspase-9 activity detection

The caspase-3 and caspase-9 activity was detected with Caspase-3 and -9 Activity Assay Kit (Solarbio, Beijing). The specific steps were conducted according to the manufacturer’s instructions. In brief, 1 × 10^6^ cells were lysed in 50 μl lysis buffer on ice for 10 mins, and centrifuged with 12,000 g at 4 °C for 10 min to collect the supernatant. Protein concentrations were detected using the Bradford method, ensuring that the protein concentration was 1–3 μg/μl. The optical density of specimen was read on a microplate reader (PerkinElmer, USA) at 405 nm. The percentage of caspase-3 and caspase-9 activity changes was calculated by the radio of OD405 of the experimental wells to that of the normal wells.

### Measurement of mitochondrial membrane potential

Changes in mitochondrial membrane potential were assessed by mitochondrial membrane potential assay kit with JC-1 (Beyotime, China). Briefly, cells were seeded at a density of 1 × 10^6^ cells per well onto 6-well plates and incubated with the medium containing JC-1 working liquid at 37 °C for 20 min. The cells treated with carbonylcyanidem-chlorophenylhydrazone (CCCP, 10uM, Beyotime, China) were used as positive control and the fluorescence images were observed using a fluorescent microscope (Leica, Germany). In addition, the cells were also collected and washed twice with PBS, and then analyzed by flow cytometry (Becton Dickinson, USA).

### TUNEL

TUNEL assay was performed on the IL-1β-treated cells using the one-step TUNEL apoptosis assay kit (Beyotime, Shanghai, China) according to the manufacturer’s instructions. Briefly, the cells were treated by TUNEL for 1 h at 37C. The cells were imaged under a fluorescent microscope (Leica, Germany) by using 488 nm excitation and 530 nm emission. The cells with green fluorescence were defined as apoptotic cells.

### Annexin V-FITC/PI double staining assay

After treatment, chondrocytes were stained with Annexin-V and propidium iodide (PI) using the Annexin-V-FITC kit (FMSAV-100, FcMACS, Nanjing, China) according to the protocol of the company. In brief, the chondrocytes were suspended in binding buffer with 1 × 10^6^ cells/ml. Afterward, 5 μl Annexin V-FITC and 10 μl PI were added and incubated in the dark for 15 min. The cell apoptotic rate was analyzed using a flow cytometer (Becton Dickinson, USA).

### Statistical analysis

Data are presented as mean ± SEM for continuous variables. Comparison between groups were conducted using the one-way ANOVA or paired t-test. A value of *P < 0.05* (two-tailed) was considered as statistically significant. All analyses and draws were performed with *Graphpad Prism 5*.

## Results

### IL-1β increased gene and Protein expression of TRPA1 in rat chondrocytes

**Fig. 1:**
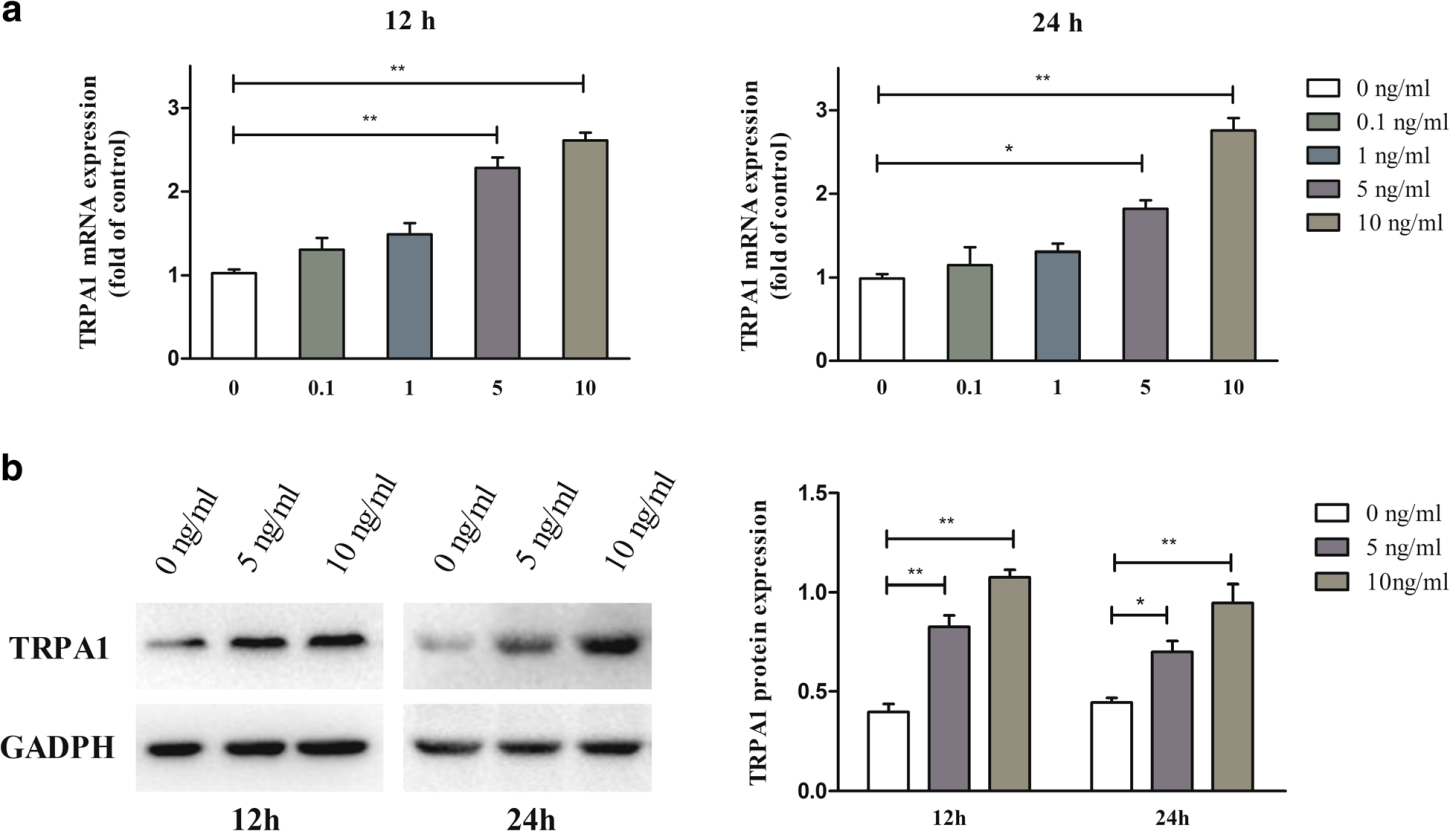
The increased expression of TRPA1 in the IL-1β-treated rat chondrocytes. **a** Rat chondrocytes were stimulated with IL-1β (0–10 ng/ml) for 12 or 24 h, and thereafter total TRPA1 mRNA were measured by PCR. **b** Chondrocytes cultures were stimulated with IL-1β (0, 5, 10 ng/ml) for 12 and 24 h, respectively, and then TRPA1 protein expression was detected with Western blot. The PCR experiment were carried out in duplicate and the quantitation of protein expression was done by densitometry analysis of the bands from three independent measurements. Values were normalized against GAPDH, and analyzed with ImageJ software. Results are expressed as mean ± SEM. ^*^p < 0.05, ^**^p < 0.01. *TRPA1 transient receptor potential ankyrin 1, IL interleukin*

In the present study, the IL-1β was found to increase gene TRPA1 expression gradually in a dose-dependent manner: the expression of TRPA1 increased up to the 10 ng/ml at 12 h or 24 h (Fig. 1a). In addition, to verify the translation of TRPA1 mRNA into protein, total protein was also detected by western blot (Fig. 1b). As a result, expression of TRPA1 protein was also increased at the 5 and 10 ng/ml in IL-1β-treated rat chondrocytes.

### IL-1β enhanced Ca^2+^ influx mediated by TRPA1 in rat chondrocytes

**Fig. 2:**
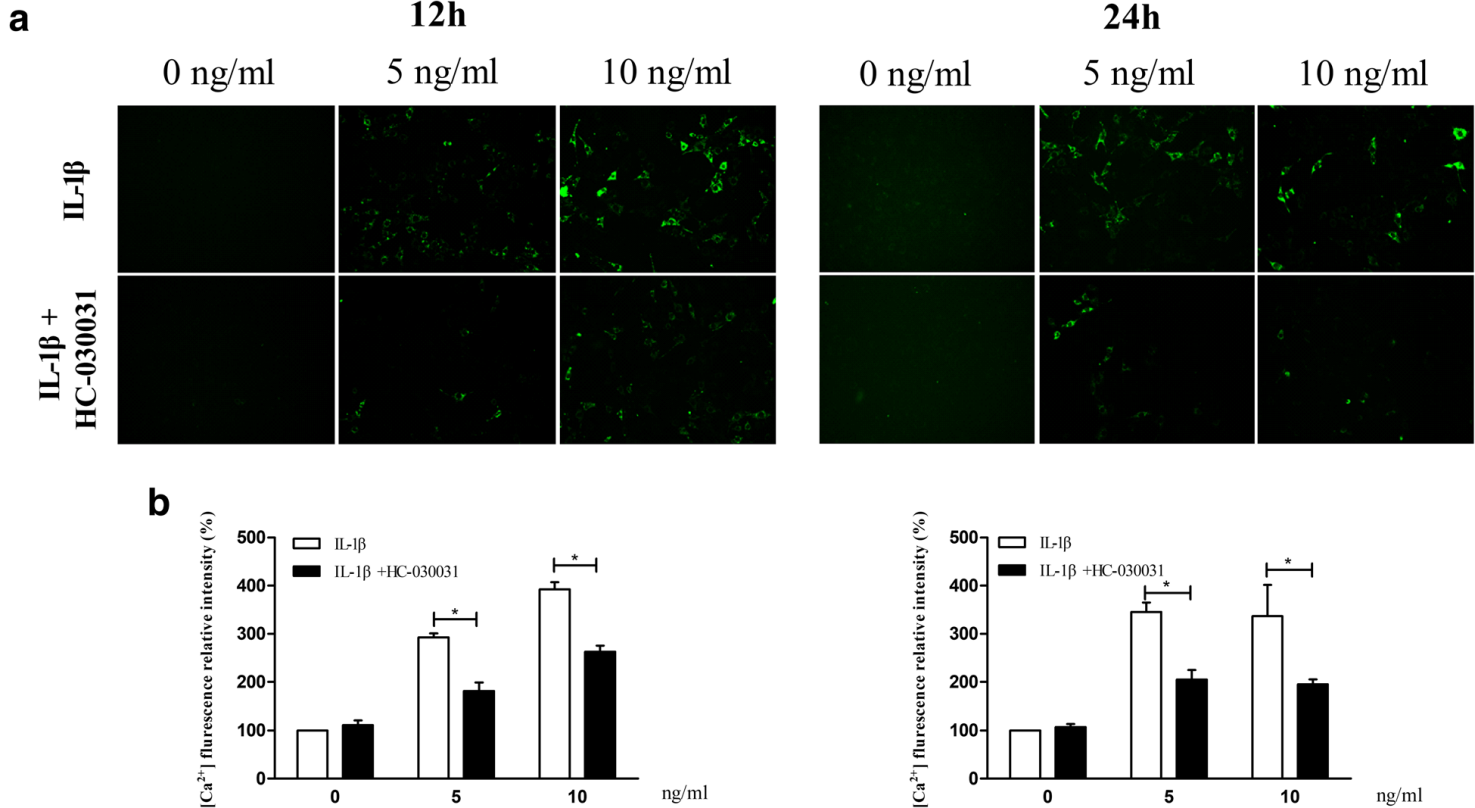
IL-1β enhanced Ca^2+^ influx mediated by TRPA1 in rat chondrocytes. Rat chondrocytes were pre-incubated with the TRPA1 antagonist (HC-030031, 100 μM) or the vehicle for 30 min and then cultured with IL-1β (0, 5, 10 ng/ml) for 12 and 24 h, respectively. The cells were loaded with Fluo-3-AM and the TRPA1-mediated Ca^2+^ influx was measured. **a** Ca^2+^ fluorescence relative intensity in different chondrocyte treatment groups (all photomicrographs are shown at × 200 magnification). **b** Bar graph showing the level of relative fluorescent intensity in each group. Relative fluorescence units refer to the ratio of mean fluorescence intensity to the background. The experiment was repeated independently three times taking duplicate measurements for each experiment. Results are expressed as mean ± SEM. ^*^p < 0.05, ^**^p < 0.01. *IL interleukin, TRPA1 transient receptor potential ankyrin 1*

To confirm the calcium load mediated by TRPA1 channel in rat chondrocytes, Ca^2+^ influx measurement was introduced in the study. The results shown that Ca^2+^ influx in the IL-1β-treated chondrocytes gradually enhanced with increasing concentrations of IL-1β (0, 5, 10 ng/ml) for 12 or 24 h (Fig. 2), while the fluorescence intensity was significantly reduced after treatment with the specific TRPA1 antagonist, which indicated that TRPA1 may have a specific role in mediating extracellular Ca^2+^ influx in the IL-1β-treated rat chondrocytes.

### IL-1β-induced apoptosis was downregulated by pharmacological inhibition of TRPA1

**Fig. 3:**
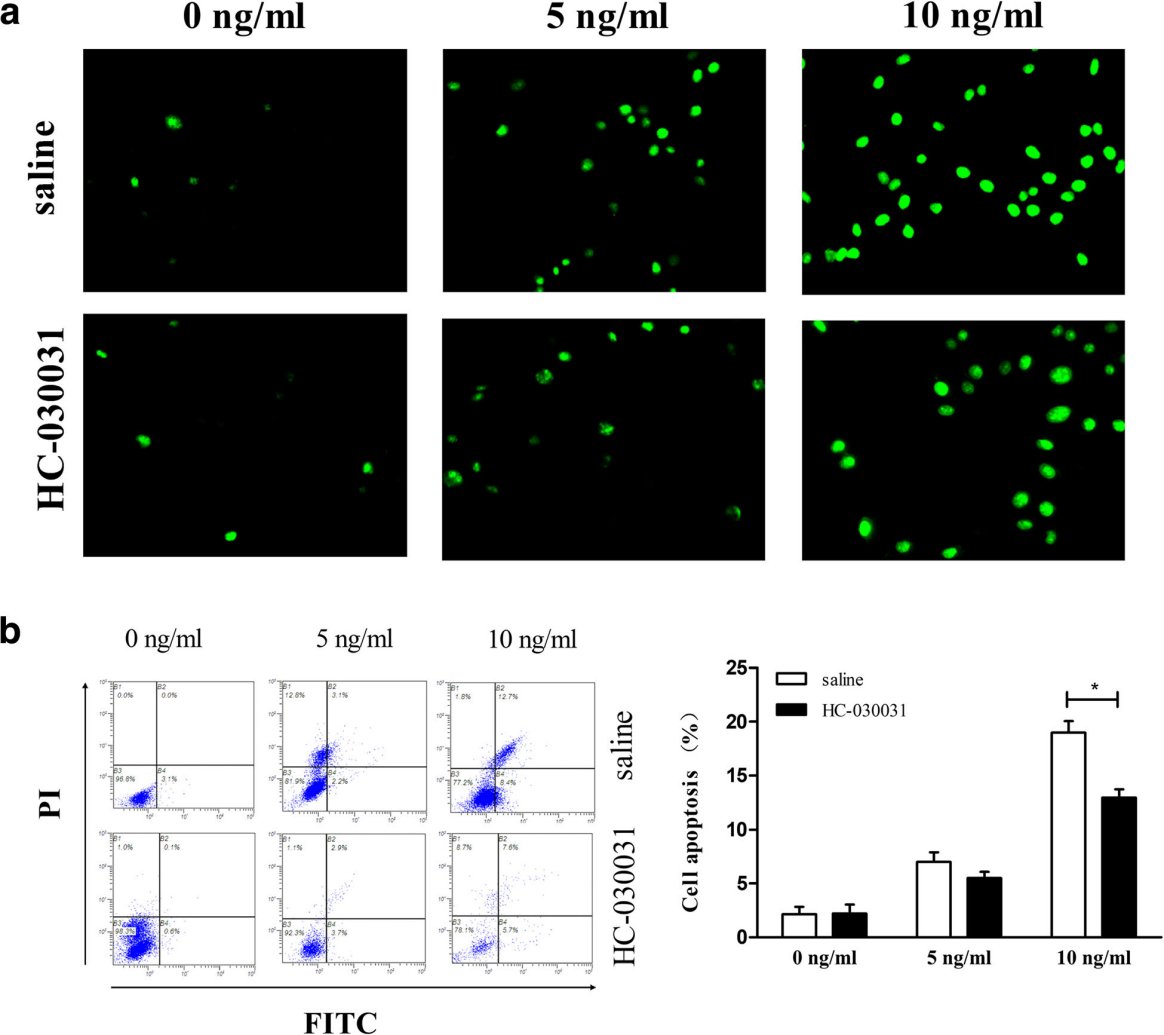
Effects of TRPA1 inhibitor on IL-1β-induced rat chondrocyte apoptosis. Rat chondrocytes were pre-incubated with the TRPA1 antagonist (HC-030031, 100 μM) or the vehicle for 30 min, and then stimulated with IL-1β (0, 5, 10 ng/ml) for 24 h. The chondrocyte apoptosis was measured by (**a**) Tunel staining and (**b**) flow cytometry. The Tunel staining was carried out in duplicate and the quantitation of apoptosis rate was repeated independently three times using flow cytometry. The results are expressed as mean ± SEM. ^*^p < 0.05, ^**^p < 0.01. *TRPA1 transient receptor potential ankyrin 1, IL interleukin*

Based on the result that IL-1β enhanced the Ca^2+^ influx mediated by TRPA1 channel, we aimed to further investigate the effect of TRPA1 inhibition on the apoptosis in the IL-1β-treated Chondrocytes. Remarkably, our results show that the percent of apoptotic cells significantly increases with increasing IL-1β concentrations, but is dramatically attenuated by pharmacological inhibition (Fig. 3).

### Effects of TRPA1 inhibition on the mitochondrial membrane potential in the IL-1β-treated chondrocytes

**Fig. 4:**
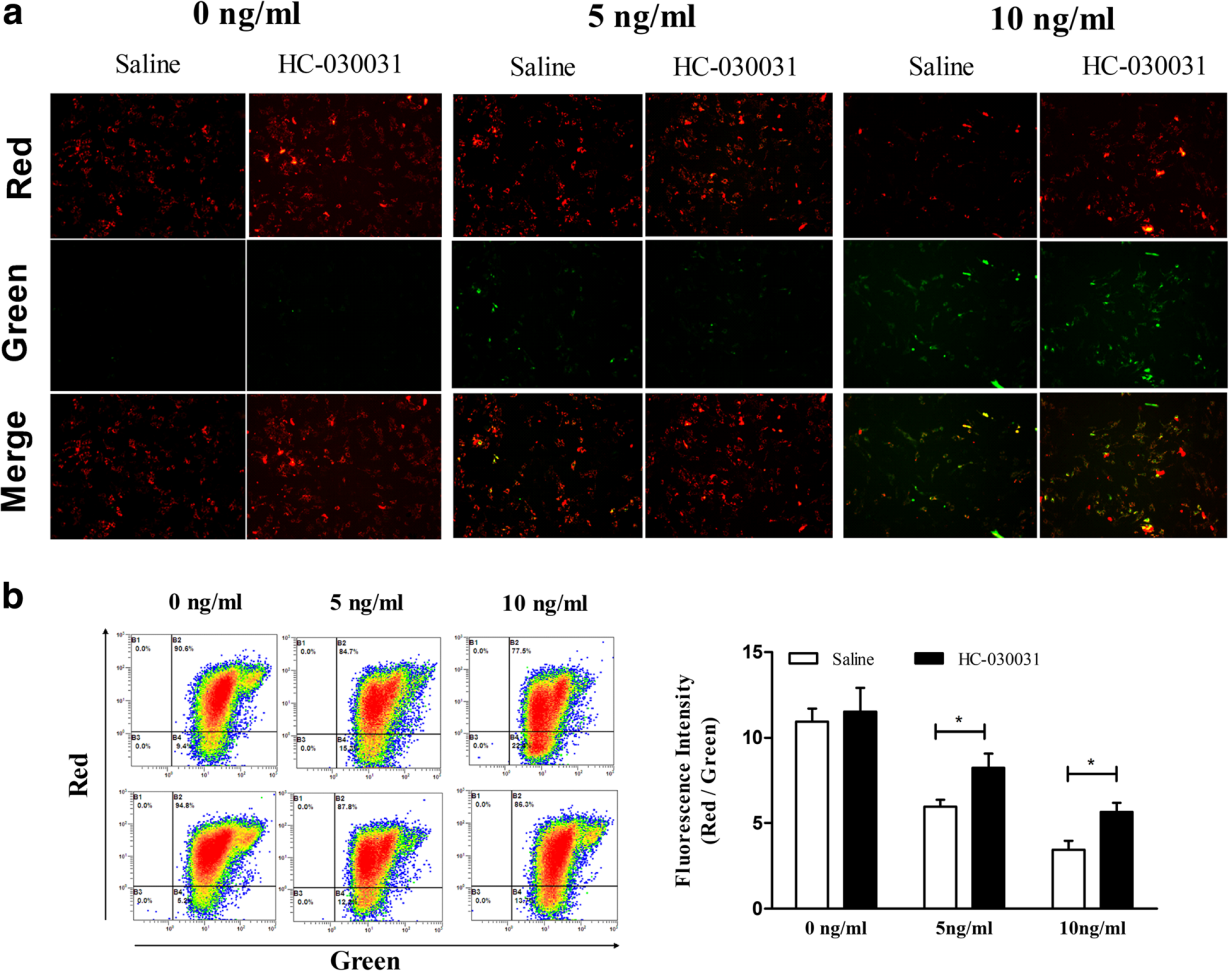
Effects of TRPA1 inhibitor on mitochondrial membrane potential in IL-1β-induced rat chondrocyte. Rat chondrocytes were pre-incubated with the TRPA1 antagonist (HC-030031, 100 μM) or the vehicle for 30 min, and then stimulated with IL-1β (0, 5, 10 ng/ml) for 24 h. **a** Mitochondrial membrane potential fluorescence images visualized by a fluorescence microscope (200×) and the experiment was carried out in duplicate. **b** Mitochondrial membrane potential detected by flowcytometry and the quantitation of mitochondrial membrane potential was repeated independently three times. The results are expressed as mean ± SEM. ^*^p < 0.05. *TRPA1 transient receptor potential ankyrin 1, IL interleukin*

Furtherly, we investigated the link between TRPA1 and mitochondrial membrane potential using JC-1 staining. As shown in Fig. 4, the mitochondrial membrane potential was reduced significantly in the IL-1β-treated cells. Treatment with TRPA1 inhibition restored the mitochondrial membrane potential (Fig. 4).

### Effects of TRPA1 inhibition on the expression of apoptosis-associated proteins in the IL-1β-treated chondrocytes

**Fig. 5:**
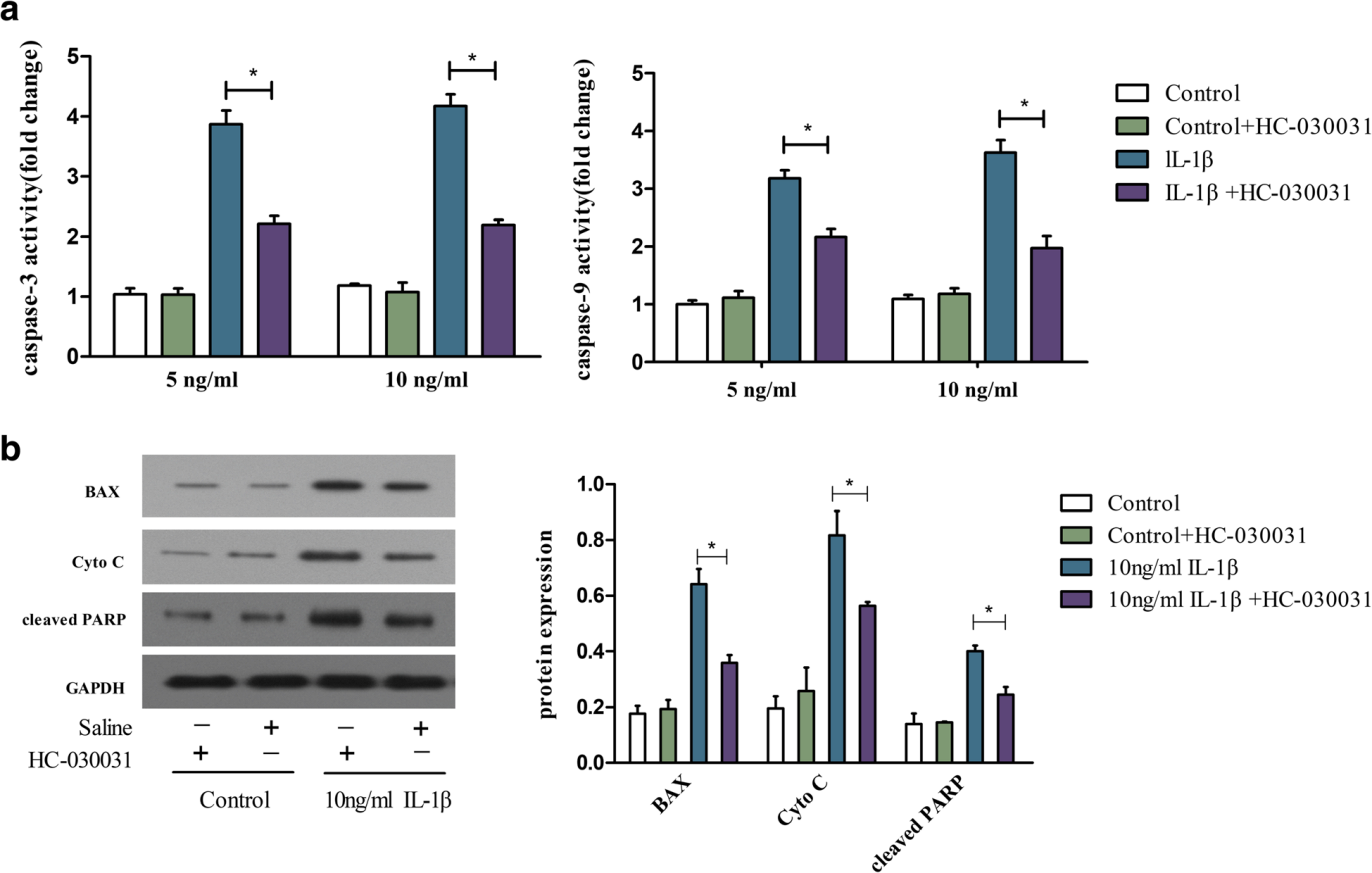
Increased calcium influx induces apoptosis by activating the mitochondrial signaling pathway in IL-1β-induced rat chondrocytes. Rat chondrocytes were pre-incubated with the TRPA1 antagonist (HC-030031, 100 μM) or the vehicle for 30 min, and then stimulated with IL-1β (5 or 10 ng/ml) for 24 h. **a** Caspase-3 and Caspase-9 activity in rat chondrocyte following various treatments. **b** The Bax, Cyto C, cleaved PARP, cleaved caspase-3 and caspase-9 protein expressions from different stimulated groups were detected by western blotting. The activity detection experiments were carried out in duplicate and the quantitation of protein expression was done by densitometry analysis of the bands from three independent measurements. Values were normalized against GAPDH, and analyzed with ImageJ software. The results are expressed as mean ± SEM. *p < 0.05. *IL interleukin, Cyto cytochrome*

The expression of BAX, cytochrome c release and cleaved PARP and the activity of caspase-3 and caspase-9 were detected to investigate the mechanisms of TRPA1 on IL-1β-induced chondrocyte apoptosis. The results showed that IL-1β significantly increased BAX, cytochrome c release, cleaved PARP protein expression (Fig. 5b), as well as the caspase-3 and 9 activity (Fig. 5a) in the chondrocytes. The treatment with TRPA1 inhibition evidently improved the abnormal expression of apoptosis-associated proteins (Fig. 5).

## Discussion

Knee OA is a common degenerative joint disease, which pathologically characterized by the progressive degeneration of articular cartilage [2]. In OA joint, the activation of innate immune system markedly contributes to an increased expression of inflammatory cytokines, which play a crucial role in the initiation, maintenance, and perpetuation of osteoarthritic cartilage destruction [26, 27]. As a key inflammatory factor, IL-1β has been widely used as a chondrocyte apoptosis-inducing agent simulating the inflammatory environment of OA in vitro [28, 29]. In the current study, through establishing a model of IL-1β-induced apoptosis, we found the IL-1β caused the increased functional expression of TRPA1 contributing to calcium overload, and inhibition of TRPA1 produced protective effect in IL-1β-induced mitochondrial dysfunction and even apoptosis in rat chondrocytes.

TRPA1, firstly described in 1991, is a nonselective cation channel, especially permeable to Ca^2+^, widely expressed in sensory neurons [30]. Recent years, emerging evidences suggested the expression of TRPA1 was also found in non-neuronal cells, synovial fibroblasts and even chondrocytes included [20, 24]. More recently, however, the inflammatory environment in OA joint, such as inflammatory cytokines, has been shown to upregulate the gene and protein expression of TRPA1. In our previous paper, we found that both LPS and its downstream factors TNF-α, IL-1β increased the gene and protein expression of TRPA1 in human osteoarthritic synovial fibroblasts in a time-dependent and dose-dependent manner [20]. Besides, according to the recent paper by Moilanen et al. [24], the gene and protein expression of TRPA1 were significantly upregulated by inflammatory factors TNF-α and IL-1β in OA human chondrocytes. In the present study, we also found that IL-1β increased the gene and protein expression of TRPA1 in rat chondrocytes in a dose-dependent manner.

Interesting, not all of them identified the functionality of TRPA1 channel although the gene expression of TRPA1 have reported in some studies. In the present study, in order to identified the increased expression of TRPA1 is functional, we measured a Ca^2+^ influx using the TRPA1 antagonist in the IL-1β-treated cells. As a result, we found the TRPA1 had a specific effect on mediating calcium influx in the IL-1β-treated rat chondrocytes, which have fairly shown the expression and activation of TRPA1 in rat chondrocytes. It is noteworthy that TRPA1 can be activated by not only numerous exogenous pungent compounds but also agents formed endogenously in inflammatory reactions, such as nitric oxide [31], prostaglandins [32], and reactive oxygen species [33]. Moreover, in OA chondrocytes, the IL-1β has the ability to stimulate the production of mediators including nitric oxide [34], prostaglandin [35], and reactive oxygen species [36], which are involved in TRPA1 channel activation. Therefore, in these inflammatory cells, IL-1β maybe firstly upregulated the gene and protein expression of TRPA1, and then the increased expression of TRPA1 channel was activated by the endogenous agonists formed in inflammatory reactions, such as nitric oxide, prostaglandins, or reactive oxygen species. However, the detailed molecular mechanisms of the activation of TRPA1 channel demands further investigation remain.

It is well known that the role of Ca^2+^ influx through Ca^2+^-selective channels has been studied extensively, such as mediating DNA fragmentation and apoptosis [8–10]. In the present paper, we also testified that, in the IL-1β-treated rat chondrocytes, calcium influx though the TRPA1channel could induce apoptosis, which agree with the previous studies indicating that TRPA1 inhibitor or deficiency could reduce the apoptosis of cardiomyocytes [37], oligodendrocytes [38], and even hippocampal neurons [39]. Apoptosis, a form of programmed cell death, is induced through two alternative pathways: extrinsic and intrinsic pathways, of which the intrinsic pathway is regulated by mitochondrial parameters [40]. Mitochondrial mediated apoptosis may initiate through the release of pro-apoptotic proteins into the cytosol due to mitochondrial dysfunction [41, 42]. Interesting, calcium influx was highlighted as being important in apoptosis attributing to its ability of inducing Ca^2+^ entry-dependent ROS production [8], mitochondrial depolarization and DNA fragmentation [9]. Our results suggested that calcium influx though the TRPA1 channel could induce mitochondrial depolarization in chondrocytes, and inhibition of TRPA1 repair the mitochondrial membrane potential. As a nonselective cation channel, TRPA1 channel also can permeate Na^+^ and K^+^ apart from Ca^2+^, and the Na^+^ and K^+^ permeation might also make cells dysfunctional [43, 44]. However, to date, the role of TRPA1-mediated Na2+ and K+ permeation in the cell apoptosis may require future research which is beyond the scope of the current study. In addition, we also found that TRPA1 inhibitor could reduce the abnormal expressions of mitochondrial pro-apoptotic proteins including BAX, cytochrome c, PARP, caspase-3 and caspase-9. The present results suggest a potential possibility that TRPA1 channel involve in the process of mitochondrial dysfunction and intrinsic pathway in IL-1β-induced apoptosis model.

**Fig. 6:**
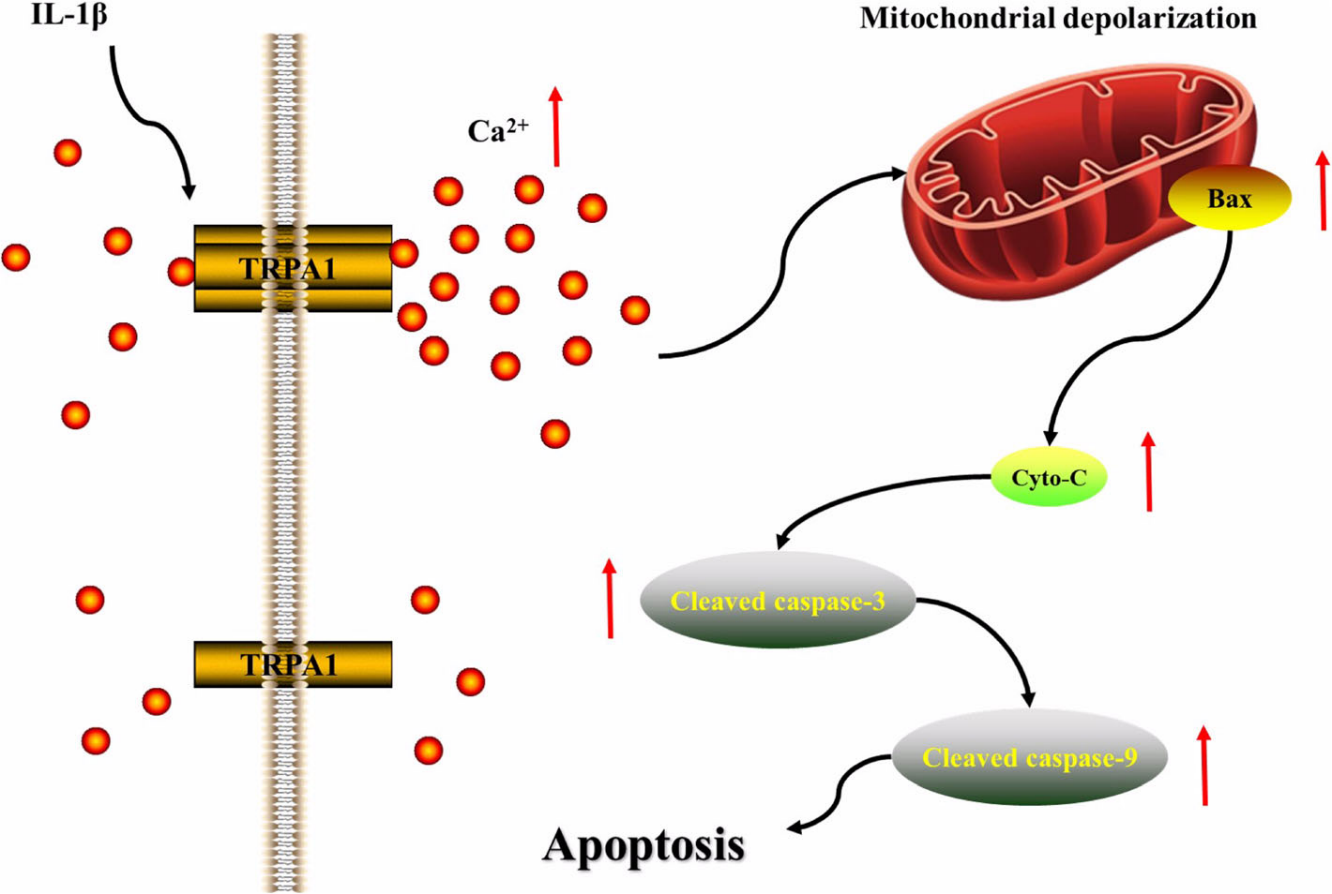
Diagram of the signaling cascade involved in the effect of TRPA1-mediated Ca^2+^ influx on apoptosis in IL-1β-induced rat chondrocytes. IL-1β caused increased functional expression of TRPA1, which contributed to massive Ca^2+^ influx. Due to cytosolic calcium overload, the mitochondrial membrane potential is significantly reduced, leading to the activation of the intrinsic pathway of apoptosis in rat chondrocytes. *TRPA1 transient receptor potential ankyrin 1, IL interleukin, Cyto cytochrome*

In conclusion, we found the IL-1β caused increased functional expression of TRPA1, which contributed to massive Ca^2+^ influx. Due to cytosolic calcium overload, the mitochondrial membrane potential is significantly reduced, leading to the activation of the intrinsic pathway of apoptosis in rat chondrocytes (Fig. 6). These findings together with previous studies suggested the TRPA1 could be an effective target for the treatment of OA.
